# High placental inositol content associated with suppressed pro-adipogenic effects of maternal glycaemia in offspring: the GUSTO cohort

**DOI:** 10.1038/s41366-020-0596-5

**Published:** 2020-05-20

**Authors:** Anne H. Y. Chu, Mya T. Tint, Hsin F. Chang, Gerard Wong, Wen Lun Yuan, Dedreia Tull, Brunda Nijagal, Vinod K. Narayana, Peter J. Meikle, Kenneth T. E. Chang, Rohan M. Lewis, Claudia Chi, Fabian K. P. Yap, Kok Hian Tan, Lynette P. Shek, Yap-Seng Chong, Peter D. Gluckman, Yung Seng Lee, Marielle V. Fortier, Keith M. Godfrey, Johan G. Eriksson, Neerja Karnani, Shiao-Yng Chan

**Affiliations:** 1 Singapore Institute for Clinical Sciences, Agency for Science, Technology and Research (A*STAR), Singapore, Singapore; 2 Department of Obstetrics and Gynaecology, Yong Loo Lin School of Medicine, National University of Singapore, National University Health System, Singapore, Singapore; 3 Department of Paediatrics, Yong Loo Lin School of Medicine, National University of Singapore, Singapore, Singapore; 4 Metabolomics Australia, Bio21 Institute of Molecular Science and Biotechnology, University of Melbourne, Melbourne, VIC Australia; 5 Metabolomics Laboratory, Baker Heart and Diabetes Institute, Melbourne, VIC Australia; 6 Department of Pathology and Laboratory Medicine, KK Women’s & Children’s Hospital, Singapore, Singapore; 7 Human Development and Health, Faculty of Medicine, University of Southampton, Southampton, UK; 8 Department of Maternal Fetal Medicine, KK Women’s and Children’s Hospital, Singapore, Singapore; 9 Liggins Institute, University of Auckland, Auckland, New Zealand; 10 Department of Diagnostic and Interventional Imaging, KK Women’s and Children’s Hospital, Singapore, Singapore; 11 MRC Lifecourse Epidemiology Unit & NIHR Southampton Biomedical Research Centre, University of Southampton & University Hospital Southampton NHS Foundation Trust, Southampton, UK; 12 Folkhalsan Research Center, Helsinki, Finland

**Keywords:** Nutrition, Endocrine system and metabolic diseases

## Abstract

**Background/Objectives:**

Maternal glycaemia promotes fetal adiposity. Inositol, an insulin sensitizer, has been trialled for gestational diabetes prevention. The placenta has been implicated in how maternal hyperglycaemia generates fetal pathophysiology, but no studies have examined whether placental inositol biology is altered with maternal hyperglycaemia, nor whether such alterations impact fetal physiology. We aimed to investigate whether the effects of maternal glycaemia on offspring birthweight and adiposity at birth differed across placental inositol levels.

**Methods:**

Using longitudinal data from the Growing Up in Singapore Towards healthy Outcomes cohort, maternal fasting glucose (FPG) and 2-hour plasma glucose (2hPG) were obtained in pregnant women by a 75-g oral glucose tolerance test around 26 weeks’ gestation. Relative placental inositol was quantified by liquid chromatography-mass spectrometry. Primary outcomes were birthweight (*n* = 884) and abdominal adipose tissue (AAT) volumes measured by neonatal MRI scanning in a subset (*n* = 262) of term singleton pregnancies. Multiple linear regression analyses were performed.

**Results:**

Placental inositol was lower in those with higher 2hPG, no exposure to tobacco smoke antenatally, with vaginal delivery and shorter gestation. Positive associations of FPG with birthweight (adjusted β [95% CI] 164.8 g [109.1, 220.5]) and AAT (17.3 ml [11.9, 22.6] per mmol glucose) were observed, with significant interactions between inositol tertiles and FPG in relation to these outcomes (*p* < 0.05). Stratification by inositol tertiles showed that each mmol/L increase in FPG was associated with increased birthweight and AAT volume among cases within the lowest (birthweight = 174.2 g [81.2, 267.2], AAT = 21.0 ml [13.1, 28.8]) and middle inositol tertiles (birthweight = 202.0 g [103.8, 300.1], AAT = 19.7 ml [9.7, 29.7]). However, no significant association was found among cases within the highest tertile (birthweight = 81.0 g [−21.2, 183.2], AAT = 0.8 ml [−8.4, 10.0]).

**Conclusions:**

High placental inositol may protect the fetus from the pro-adipogenic effects of maternal glycaemia. Studies are warranted to investigate whether prenatal inositol supplementation can increase placental inositol and reduce fetal adiposity.

## Introduction

Increasing maternal glycaemia in pregnancy is accompanied by rising risks of fetal macrosomia and excessive fetal adiposity across the glycaemia continuum [1, 2]. Fetal macrosomia is associated with poor perinatal outcomes such as shoulder dystocia, birth trauma, caesarean section delivery, neonatal hypoglycaemia, and perinatal death [3]. Importantly, fetal macrosomia and accompanying increased adiposity have been linked with long-term offspring metabolic risks, including obesity, type 2 diabetes, metabolic syndrome and cardiovascular disease [4].

Pedersen hypothesised that fetal macrosomia in diabetic mothers is due to the anabolic and lipogenic effects of fetal hyperinsulinemia resulting from increased maternal-fetal glucose transfer [5]. Since then, there have been findings that infants born to women with gestational diabetes mellitus (GDM) had greater fat mass compared with infants born to women without GDM, even in neonates of normal birthweight [6]. This has led to the hypothesis that transplacental transfer of other nutrients such as lipids may also be dysregulated in GDM pregnancies. Thus, the placenta, which facilitates maternal-fetal nutrient-waste exchange as well as the bilateral maternal-fetal signals to optimise nutritional supply to the developing fetus, remains a major focus for research in considering how maternal hyperglycaemia and metabolic dysfunction generate fetal pathophysiology.

Inositol supplementation from early pregnancy has been proposed to prevent GDM in women at risk, including those with a family history of diabetes [7], polycystic ovary syndrome [8], and a high body mass index (BMI) [9, 10]. Several relatively small randomised control trials (RCT) have shown inconsistent results in relation to the incidence of GDM [11, 12] and macrosomia [13]. Larger trials are underway to definitively determine its efficacy.

Inositol is a natural 6-carbon carbohydrate abundant in fruits, grains, and nuts. It is also produced endogenously, mainly in the kidneys [14]. Inositol is a key constituent of many second messenger signalling compounds and important membrane phospholipids. One key function of inositol is to promote insulin signal transduction, thus it acts as an insulin sensitiser to improve maternal metabolic status [15].

There is limited knowledge about inositol uptake and passage through the placenta, the influence of hyperglycaemia on these processes, and the implications of altered placental inositol biology on the fetus. A short-term in vivo stable isotope study in full-term pregnancies showed very little maternal to fetal transfer of inositol [16]. Maternal inositol may indirectly influence fetal adiposity through its insulin-sensitising effects in maternal tissues to promote normoglycaemia. With some suggestions that inositol and its derivatives accumulate in human placenta [17] and are therefore likely to play an important role in this tissue, alternatively, we hypothesised that increasing placental inositol content could alter fetoplacental physiology to attenuate the adipogenic effects of maternal hyperglycaemia in the offspring.

Using a longitudinal mother-offspring cohort study, we quantified placental inositol content in term singleton pregnancies and examined: (i) the participant characteristics and factors related to placental inositol content, (ii) the associations of placental inositol with neonatal birthweight and adiposity, and (iii) the associations of maternal glycaemia with neonatal birthweight and adiposity across different placental inositol levels.

## Materials and methods

### Study population

The present study is embedded in the Growing Up in Singapore Towards healthy Outcomes (GUSTO) study which was designed to examine the role of antenatal and early life factors in the developmental origins of health and disease. Details of the GUSTO cohort study are available elsewhere [18]. Briefly, pregnant women of the three major Singaporean ethnic groups, Chinese, Malays and Indians (aged ≥18 years) in their first trimester were recruited from Singapore’s two major public maternity units, the KK Women’s and Children’s Hospital and the National University Hospital between 2009 and 2010. Eligible women were recruited around 10–14 weeks’ gestation (*n* = 1247), with written informed consent obtained. The GUSTO study was approved by the National Health Care Group Domain Specific Review Board (reference D/09/021) and the SingHealth Centralized Institutional Review Board (reference 2009/280/D).

### Baseline characteristics

Information on self-reported maternal age and parity was confirmed from medical records. Parity was categorised as nulliparous or parous. Self-reported ethnicity was categorised as Chinese or non-Chinese (Malay and Indian). Pre-pregnancy BMI (kg/m^2^) was derived from reported pre-pregnancy weight and categorised using the WHO criteria for Asian populations: underweight (<18.5), normal (18.5–22.9), overweight (23.0–27.4) and obese (≥27.5) [19]. Tobacco smoke exposure was categorised as “Yes” for those with detectable plasma cotinine around 26 weeks’ gestation or who self-reported active or passive smoking, and “No” for the others [20].

### Maternal glycaemia during pregnancy

Maternal fasting plasma glucose (FPG) and 2-hour plasma glucose (2hPG) levels were assessed by a 75-g oral glucose tolerance test at around 26 weeks’ gestation (mean [standard deviations, SD]: 26.9 [2.0] weeks’ gestation). GDM was diagnosed using the 1999 WHO criteria (either FPG ≥ 7.0 mmol/L or 2hPG ≥ 7.8 mmol/L), the criteria used in clinical practice at the time the GUSTO women were pregnant. Details of GDM treatment were collected from medical records and classified as diet-control only, with insulin or no treatment.

### Antenatal complications and pregnancy outcomes

Information was obtained from medical records. Hypertensive disorders were classified as pre-eclampsia or pregnancy-induced hypertension defined as new-onset BP ≥ 140/90 mmHg on at least two occasions more than four hours apart occurring after 20 weeks’ gestation, with pre-eclampsia cases also displaying proteinuria ≥300 mg/24 h or a dipstick reading of ≥1+, or abnormal liver function or elevated uric acid, or as chronic (pre-pregnancy) hypertension. Gestational age at birth was calculated using crown-rump length measured by ultrasound scan at 7–12 weeks (Hadlock curves) [21] and classified into two categories: early-term births (37 completed weeks to 38 weeks and 6 days) and term births (39 completed weeks to 41 weeks and 6 days). Mode of delivery was classified as non-labour caesarean section, intrapartum caesarean section, or vaginal delivery. Timing for collection of placental tissues after delivery was categorised as ≤30, 31–60, 61–90, or ≥91 min.

### Offspring birthweight and adiposity measurements at birth

Outcome measures of birthweight and adiposity at birth were used as continuous variables. Birthweight was expressed in g or as gestational age- and sex-specific z-scores, calculated based on locally-derived references [22]. Magnetic resonance imaging (MRI) was performed within two weeks of delivery (mean [SD]: 9.5 [2.8] days) in a subset of neonates (with a birthweight ≥2000 g) whose mothers consented to the procedure. Volumes of abdominal adipose tissue (AAT) compartments, superficial (sSAT), deep subcutaneous (dSAT) and internal adipose tissue (IAT) were segmented and quantified from MRI images using an in-house semi-automated quantitative analysis algorithm with MATLAB 7.13 software (The MathWorks Inc., Natick, Massachusetts, US) and optimised manually as previously described [23]. Total AAT (TAAT) was calculated by summing sSAT, dSAT and IAT volumes.

### Placental samples and inositol quantification

Placentae were collected post-delivery and biopsies were taken from the peri-umbilical region and stored frozen at −80°C until batch analysis. A single biopsy of the villous placenta from each participant was used. Approximately 200 mg of placental tissue was homogenised in 750 µl of milli-Q water and the homogenate made up to 1.0 ml with water. From this, 120 µL was used and 480 µl of ice-cold Acetonitrile containing 4 µM ^13^C-sorbitol & 4 µM ^13^C,^15^N-l-Valine & ^13^C-l-Leucine was added. The sample was vortexed and mixed for 10 min at 4 ˚C on the thermomixer at 950 RPM to aid lysis, then centrifuged at 14000 RPM for 10 min at 4 ˚C to remove cellular debris. Liquid chromatography-mass spectrometry (LC-MS) analysis of polar metabolites was performed as we have previously described [24]. Briefly, metabolite separation was performed on an Agilent Technologies 1260 High-performance liquid chromatography system (Agilent Technologies, Santa Clara, US) attached to a SeQuant ZIC–pHILIC column (150 × 4.6 mm). Mass spectrometry analysis was performed on an Agilent Technologies 6545 A Quadrupole Time of Flight (TOF) with published data acquisition parameter [24]. Inositol identification was based on the retention time and molecular mass matching an authentic myo-inositol standard (>99% purity; Sigma-Aldrich CAS 87-89-8, product number I-5125). A full resolution of all inositol isomers was not undertaken and measurements were regarded as a composite of all water-soluble inositol present. Peak area integration was performed using MassHunter TOF Quantitative Analysis software (version B.07.00, Agilent Technologies) and Metabolomics Australia in-house data processing pipeline. Pooled placenta quality control (QC) samples were used and incorporated once every 10 samples in the run. Inositol levels were normalised to both the ^13^C-l-Leucine standard and the protein concentration of the homogenate (quantified by Bradford assay). The normalised inositol levels were subsequently median-centred across the batches using the QCs as the basis for aligning the medians.

### Statistical methods

Only spontaneously conceived singleton pregnancies delivering from 37 completed weeks’ gestation onwards with available placental biopsies for inositol quantification were included in this study (*n* = 888). After exclusion of three outliers which had an inositol >4 SDs from the mean and one extreme outlier from the mean birthweight (8.65 SDs), 884 cases were included in the analysis. A subsample of children with valid results from abdominal MRI (*n* = 262) were also analysed.

Data are expressed as means (SD) or *n* (%). Relative levels of placental inositol were normally distributed and standardised to z-scores. One-way ANOVA was used to compare differences in mean inositol z-score of the groups categorised by maternal and neonatal factors. Tukey’s post-hoc tests were used to compare group means where relevant. Pearson’s linear correlation coefficient (*r*) was used to examine the relation between inositol and maternal glycaemia (FPG and 2hPG).

Multiple linear regression analysis was performed. The covariates adjusted for were factors associated with placental inositol from univariate analysis, and alongside other potential covariates known to be associated with metabolism and placental function (as continuous variables: maternal age, pre-pregnancy BMI, gestational age; as categorical variables with classifications as described earlier: ethnicity, parity and neonatal sex).

We next assessed whether there was a linear or quadratic (U-shaped) association of placental inositol and birthweight, with adjustment for covariates based on literature (as continuous variables: maternal age, pre-pregnancy BMI, FPG, gestational age; as categorical variables: ethnicity, parity, tobacco smoke exposure, neonatal sex) and with additional technical adjustments for factors influencing placental inositol, i.e. mode of delivery and timing of placental collection.

Then, we determined the association of maternal FPG with birthweight, birthweight z-score and adiposity. Next, we examined the potential modulating effect of inositol on these associations between maternal glycaemia and birthweight or adiposity. Stratification into inositol tertiles (lowest, middle and highest inositol groups) was performed based on the entire data set (*n* = 884). The same inositol thresholds were used to determine inositol groups in the smaller MRI data set (*n* = 262). We included an interaction term between inositol tertiles and glycaemia adjusting for covariates (listed above). If the interaction term was significant, we then derived adjusted beta coefficients for the associations between glycaemia and birthweight/adiposity by inositol tertiles. In abdominal adiposity analyses, we further adjusted for birthweight (to control for overall neonatal size) and neonatal age on the day of MRI. In addition to performing the analysis on the whole sample, to assess whether GDM status influenced the magnitude of the glycaemia-birthweight/AAT associations in different placental inositol tertiles, we conducted further stratified analysis separating normoglycaemic cases from GDM cases. We also performed tests of interaction for ethnicity and neonatal sex for these associations in the whole sample. Only cases with complete datasets were used and missing values were not imputed.

To examine the robustness of our results, sensitivity analyses were conducted by excluding: (i) cases of possible pre-existing diabetes (FPG ≥ 7.0 mmol/L or 2hPG ≥ 11.1 mmol/L; *n* = 6), (ii) cases who received insulin treatment for GDM or possible pre-existing diabetes (*n* = 10), (iii) cases with hypertensive disorders (*n* = 44), and (iv) infants born small-for-gestational-age (<10th centile, *n* = 73). Further technical adjustment of inositol values for mode of delivery and timing of placental collection was also performed. A two-sided α-level of 5% was considered significant. Stata 15 software (StataCorp LP, TX) was used for statistical analyses.

## Results

Participant characteristics and the corresponding mean placental inositol z-score for each sub-category of all the factors examined are presented in Table 1. Lower placental inositol was associated with the antenatal factors of increasing maternal 2hPG at mid-gestation and absence of tobacco smoke exposure, the peri-partum factor of lower gestational age at birth and the pre-analytical factor of longer timing to placental collection. There was also a trend of lower placental inositol with vaginal births. After mutual adjustment for the factors identified to be associated with placental inositol differences and for relevant covariates (Table 2), placental inositol remained higher among women exposed to tobacco smoke compared with the unexposed. Placenta from vaginal deliveries now had a significantly lower inositol compared with the non-labour caesarean section group. Placental inositol was also modestly lower with each mmol/L increase in 2hPG and marginally higher with each incremental day of gestation.

**Table 1:** Characteristics of study participants and relative levels of placental inositol content.

	*N* (%)^a^	Placental Inositol z-score, Mean (SD)	*P* value^b^
Maternal age (years)			0.414
<25	98 (11.1)	−0.11 (1.13)	
≥25 & <35	586 (66.3)	0.03 (1.00)	
≥35	200 (22.6)	−0.03 (0.94)	
Ethnicity			0.276
Chinese	530 (60.0)	−0.03 (0.96)	
Non-Chinese	354 (40.0)	0.04 (1.06)	
Parity			0.304
Nulliparous	386 (43.7)	−0.04 (1.01)	
Parous	498 (56.3)	0.03 (0.99)	
Pre-pregnancy BMI (kg/m^2^)			0.361
Underweight (<18.5)	98 (11.1)	−0.12 (0.99)	
Normal (≥18.5 & <23)	427 (48.3)	0.01 (1.00)	
Overweight (≥23 & <27.5)	196 (22.2)	0.05 (1.08)	
Obese (≥27.5)	105 (11.9)	−0.11 (0.88)	
Missing	58 (6.5)		
Tobacco smoke exposure			0.002
No	450 (50.9)	−0.09 (0.98)	
Yes	348 (39.4)	0.13 (1.03)	
Missing	86 (9.7)		
Hypertensive disorders			0.559
No gestational hypertension	833 (94.2)	−0.01 (1.00)	
Pre-eclampsia/Eclampsia	18 (2.1)	0.16 (1.00)	
Non-proteinuric pregnancy-induced hypertension	25 (2.8)	0.05 (1.09)	
Chronic hypertension	2 (0.2)	−0.86 (0.82)	
Missing	6 (0.7)		
Gestational age at birth (weeks)			0.010
37^+0^ to 38^+^ [6] weeks	393 (44.5)	−0.10 (1.06)	
39^+0^ weeks or more	491 (55.5)	0.08 (0.94)	
Neonatal sex			0.454
Male	471 (53.3)	0.02 (1.02)	
Female	413 (46.7)	−0.03 (0.98)	
Mode of delivery			0.057
Non-labour caesarean section	117 (13.2)	0.17 (0.97)	
Intrapartum caesarean section	137 (15.5)	0.59 (0.87)	
Vaginal delivery	630 (71.3)	−0.04 (1.03)	
Timing of placental collection after delivery (minutes)			0.037
≤30	110 (12.4)	0.20 (1.09)	
31–60	433 (49.0)	0.03 (0.96)	
61–90	160 (18.1)	−0.10 (1.01)	
≥91	89 (10.1)	−0.15 (0.97)	
Missing	92 (10.4)		
GDM (WHO 1999 criteria)			0.127
No	691 (78.2)	0.02 (0.99)	
Yes	154 (17.4)	−0.12 (1.04)	
Missing	39 (4.4)		
		Pearson’s *r*	*P* value
FPG (mmol/L)	845 (95.6)	−0.01	0.671
2hPG (mmol/L)	845 (95.6)	−0.08	0.023

*BMI* body mass index, *FPG* fasting plasma glucose, *GDM* gestational diabetes mellitus, *2hPG* 2-hour plasma glucose, *WHO* World Health Organization.

^a^Based on available data.

^b^One-way ANOVA test of mean difference in inositol z-score between groups.

**Table 2:** Factors showing significant or borderline significant association with placental inositol.

Categorical variable	Unadjusted β (95% CI) placental inositol z-score (SDs)	*P* value	Adjusted β (95% CI)^a^ placental inositol z-score (SDs)	*P* value
Tobacco smoke exposure				
No	Reference		Reference	
Yes	0.22 (0.08, 0.36)	0.002	0.24 (0.08, 0.40)	0.003
Mode of delivery				
Non-labour caesarean section	Reference		Reference	
Intrapartum caesarean section	−0.11 (−0.36, 0.14)	0.388	−0.15 (−0.44, 1.35)	0.299
Vaginal delivery	−0.21 (−0.41, −0.14)	0.036	−0.29 (−0.52, −0.06)	0.013
Timing of placental collection after delivery (minutes)				
≤30	Reference		Reference	
31–60	−0.18 (−0.39, 0.03)	0.094	−0.18 (−0.41, 0.05)	0.133
61–90	−0.30 (−0.54, −0.06)	0.013	−0.29 (−0.55, −0.02)	0.033
≥91	−0.35 (−0.63, −0.07)	0.013	−0.22 (−0.53, 0.08)	0.148
Continuous variable	Unadjusted β (95% CI) Placental inositol SDs/unit increase	*P* value	Adjusted β (95% CI)^a^ Placental inositol SDs/unit increase	*P* value
2hPG (mmol/L)	−0.05 (−0.10, −0.01)	0.021	−0.06 (−0.12, −0.01)	0.028
Gestational age at birth (days)	0.01 (0, 0.02)	0.026	0.01 (0, 0.03)	0.008

*2hPG* 2-hour plasma glucose.

^a^Adjusted for maternal age, ethnicity, parity, pre-pregnancy BMI, neonatal sex, and mutually adjusted for tobacco smoke exposure, gestational age, mode of delivery, timing of placental collection after delivery, and 2hPG at mid-gestation.

There was no association between placental inositol and birthweight (*P* = 0.474) after adjustment for covariates and technical factors (mode of delivery and timing of placental collection). However, there were significant interactions between the influences of placental inositol tertiles and maternal FPG (but not 2hPG) on birthweight (Table 3) and AAT compartment volumes (Table 4), hence subsequent analyses were performed using inositol tertiles. There was no difference in maternal age, ethnicity, parity and BMI along with other characteristics across the groups of inositol tertiles (Table 5).

**Table 3:** Adjusted associations of maternal mid-gestation FPG with birthweight and birthweight z-score stratified by placental inositol tertiles.

FPG (mmol/L)		Birthweight (g)^a^		Birthweight z-score (SDs)^b^	
*P* for interaction^c^		0.028		0.012	
Tertiles of inositol	Inositol z-score, range	β^d^ (95% CI)	*P* value	β^d^ (95% CI)	*P* value
Whole sample					
Lowest (*n* = 245^e^)	−3.337, −0.470	174.2 (81.2, 267.2)	<0.001	0.6 (0.3, 0.9)	<0.001
Middle (*n* = 240^e^)	−0.469, 0.336	202.0 (103.8, 300.1)	<0.001	0.7 (0.4, 1.0)	<0.001
Highest (*n* = 239^e^)	0.337, 3.990	81.0 (−21.2, 183.2)	0.120	0.2 (−0.1, 0.6)	0.177
Cases of normoglycaemia^f^					
Lowest (*n* = 189^e^)	−3.337, −0.470	175.0 (54.4, 295.6)	0.005	0.6 (0.2, 1)	0.003
Middle (*n* = 198^e^)	−0.469, 0.336	136.9 (1.1, 272.7)	0.048	0.5 (0, 0.9)	0.041
Highest (*n* = 199^e^)	0.337, 3.914	114.0 (−24.1, 252)	0.105	0.3 (−0.1, 0.7)	0.193
Cases of gestational diabetes (GDM)^g^					
Lowest (*n* = 56^e^)	−3.096, −0.481	239.3 (64.0, 414.6)	0.009	0.9 (0.3, 1.5)	0.002
Middle (*n* = 42^e^)	−0.436, 0.315	181.9 (4.3, 359.4)	0.045	0.6 (0, 1.1)	0.040
Highest (*n* = 40^e^)	0.355, 3.990	−78.9 (−308.2, 150.5)	0.488	−0.4 (−1.2, 0.4)	0.280

*FPG* fasting plasma glucose, *GDM* gestational diabetes mellitus.

^a^Adjusted for maternal age, ethnicity, parity, pre-pregnancy BMI, tobacco smoke exposure, gestational age, and neonatal sex.

^b^Adjusted for maternal age, ethnicity, parity, pre-pregnancy BMI, and tobacco smoke exposure.

^c^Interaction between placental inositol tertiles and FPG on birthweight/birthweight z-score with adjustment for covariates in the whole sample.

^d^Beta (β) represents the change in g or SDs per mmol/L increase in FPG.

^**e**^Only cases with full data sets available are presented.

^f^Defined as those with normal oral glucose tolerance test by the WHO 1999 criteria.

^g^Abnormal oral glucose tolerance test by the WHO 1999 criteria (FPG ≥ 7.0 mmol/L or 2hPG ≥ 7.8 mmol/L).

**Table 4:** Adjusted associations between maternal mid-gestation FPG and neonatal abdominal adiposity stratified by placental inositol tertiles.

FPG (mmol/L)		sSAT (ml)		dSAT (ml)		IAT (ml)		TAAT (ml)	
*P* for interaction^a^		0.021		0.015		0.016		0.004	
Tertiles of inositol	Inositol z-score, range	β^b^ (95% CI)	*P* value	β^b^ (95% CI)	*P* value	β^b^ (95% CI)	*P* value	β^b^ (95% CI)	*P* value
All in MRI subset									
Lowest (*n* = 80^c^)	−3.337, −0.473	12.0 (6.2, 17.8)	<0.001	3.5 (1.8, 5.3)	<0.001	5.4 (3.5, 7.4)	<0.001	21.0 (13.1, 28.8)	<0.001
Middle (*n* = 65^c^)	−0.469, 0.336	12.1 (5.7, 18.5)	0.004	3.3 (1.1, 5.5)	0.002	4.3 (1.4, 7.5)	0.009	19.7 (9.7, 29.7)	<0.001
Highest (*n* = 74^c^)	0.342, 3.610	1.6 (−4.9, 8.0)	0.625	−0.3 (−2.6, 2.0)	0.805	−0.5 (−3.8, 2.9)	0.770	0.8 (−8.4, 10.0)	0.862
Cases of normoglycaemia with valid MRI data^d^									
Lowest (*n* = 64^c^)	−3.337, −0.473	12.5 (4.6, 20.4)	0.003	3.2 (0.6, 5.8)	0.016	5.6 (2.5, 8.6)	0.001	21.3 (10.2, 32.3)	<0.001
Middle (*n* = 52^c^)	−0.469, 0.336	5.7 (−5.5, 16.9)	0.307	0 (-3.9, 3.9)	0.997	−2.1 (−6.8, 2.6)	0.378	3.7 (−13.3, 20.6)	0.666
Highest (*n* = 66^c^)	0.342, 3.610	−1.7 (−9.9, 6.5)	0.685	−1.4 (−4.4, 1.6)	0.340	−2 (−6.4, 2.4)	0.378	−5.0 (−16.8, 6.7)	0.394

Adjusted for maternal age, ethnicity, parity, pre-pregnancy BMI, tobacco smoke exposure, gestational age, neonatal sex, birthweight, and age on MRI day.

*dSAT* deep subcutaneous adipose tissue, *FPG* fasting plasma glucose, *IAT* internal adipose tissue, *sSAT* superficial subcutaneous adipose tissue, *TAAT* total abdominal adipose tissue (sum of sSAT, dSAT and IAT).

^a^Interaction between placental inositol tertiles and FPG on neonatal abdominal adiposity with adjustment for covariates among all in the MRI subset.

^b^Beta (β) represents the change in ml per mmol/L increase in FPG.

^c^Only cases with full data sets available are presented.

^d^Defined as those with normal oral glucose tolerance test by the WHO 1999 criteria.

**Table 5:** Participant characteristics across the placental inositol tertile groups.

	Groups of placental inositol tertiles			
	Lowest	Middle	Highest	*P* value^a^
Inositol z-score, range	−3.337, −0.470	−0.469, 0.336	0.337, 3.990	
Maternal age (years)	30.9 ± 5.0	31.2 ± 5.2	31.3 ± 4.9	0.590
Ethnicity				0.601
Chinese	171 (58.0)	183 (62.0)	176 (59.9)	
Non-Chinese	124 (42.0)	112 (38.0)	118 (40.1)	
Parity				0.400
Nulliparous	138 (46.8)	126 (42.7)	122 (41.5)	
Parous	157 (53.2)	169 (57.3)	172 (58.5)	
Pre-pregnancy BMI (kg/m^2^)	22.5 ± 4.6	22.6 ± 4.1	22.3 ± 3.9	0.690
Tobacco smoke exposure				0.167
No	163 (55.3)	153 (51.9)	134 (45.6)	
Yes	103 (34.9)	117 (39.7)	128 (43.5)	
Missing	29 (9.8)	25 (8.5)	32 (10.9)	
Gestational age at birth (weeks)	39.0 ± 1.0	39.1 ± 1.0	39.2 ± 1.0	0.090
Neonatal sex				0.745
Male	152 (51.5)	161 (54.6)	158 (53.7)	
Female	143 (48.5)	134 (45.4)	136 (46.3)	
Mode of delivery				0.137
Non-labour caesarean section	32 (10.9)	41 (13.9)	44 (15)	
Intrapartum caesarean section	38 (12.9)	55 (18.6)	44 (15)	
Vaginal delivery	225 (76.3)	199 (67.5)	206 (70.1)	
Timing of placental collection after delivery (minutes)				0.116
≤30	33 (11.2)	31 (10.5)	46 (15.7)	
31–60	132 (44.8)	150 (50.9)	151 (51.4)	
61–90	56 (19.0)	56 (19.0)	48 (16.3)	
≥91	37 (12.5)	31 (10.5)	21 (7.1)	
Missing	37 (12.5)	27 (9.2)	28 (9.5)	
GDM (WHO 1999 criteria)				0.053
No	218 (73.9)	234 (79.3)	239 (81.3)	
Yes	64 (21.7)	47 (15.9)	43 (14.6)	
Missing	13 (4.4)	14 (4.8)	12 (4.1)	
GDM Treatment				0.941
Diet-only	57 (20.2)	42 (15.0)	38 (13.5)	
Insulin	4 (1.4)	3 (1.1)	4 (1.4)	
None	3 (1.1)	2 (0.7)	1 (0.4)	
FPG (mmol/L)	4.3 ± 0.5	4.4 ± 0.5	4.3 ± 0.4	0.069
2hPG (mmol/L)	6.6 ± 1.5	6.5 ± 1.4	6.4 ± 1.4	0.226

Data presented as mean **±** SD or *n* (%).

*BMI* body mass index, *FPG* fasting plasma glucose, *GDM* gestational diabetes mellitus, *2hPG* 2-hour plasma glucose.

^a^Chi-squared test (for categorical variables) and Fisher’s exact test (for categorical variables with cells having an expected frequency of five or less) were used to analyse categorical data. One-way ANOVA was used to compare the means across groups.

**Fig. 1:**
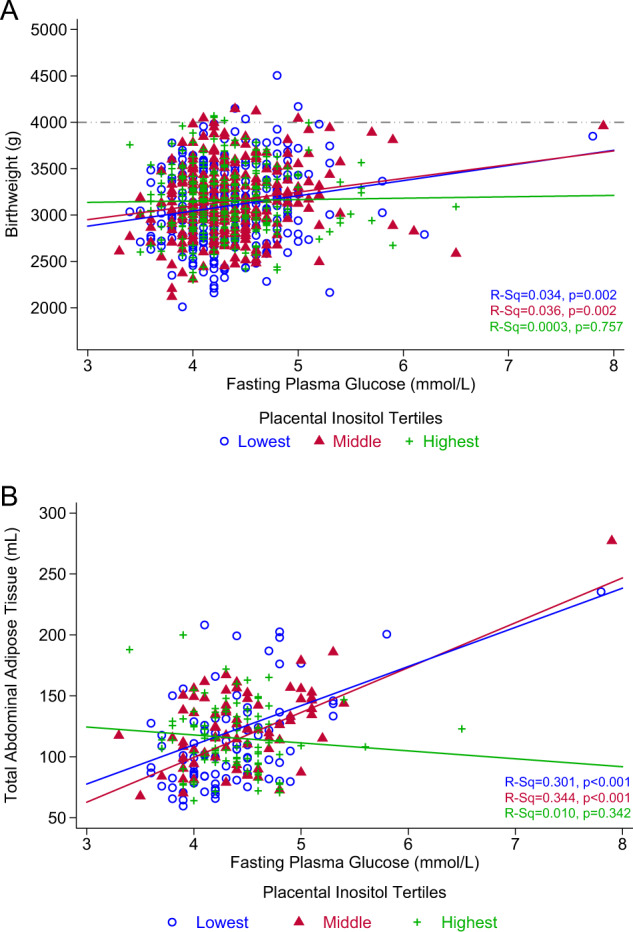
Maternal fasting plasma glucose with birthweight and neonatal adiposity, stratified by placental inositol tertiles. Comparison of maternal mid-gestation fasting plasma glucose with: (**a**) birthweight (*n* = 884) and (**b**) MRI-derived total abdominal adipose tissue (*n* = 262). Pearson’s correlation (R-Sq) is presented, representing data with no adjustments for covariates. The dash-dot-dot line represents fetal macrosomia with abirthweight  ≥ 4000 g.

Overall, the mean (SD) birthweight of the neonates was 3139.3 g (391.5). After adjusting for covariates, FPG was associated with increasing birthweight (β = 164.8 g per mmol/L glucose, 95% CI: 109.1–220.5) and birthweight z-score (β = 0.5 SDs per mmol/L glucose, 95% CI: 0.4–0.7). When stratified by inositol tertiles, the positive associations between FPG and birthweight were observed only among cases within the lowest and middle tertiles in both unadjusted (Fig. 1a) and adjusted analyses (Table 3). Interestingly, among cases in the highest inositol tertile, there was no significant association between FPG and birthweight. In analysis further stratifying by GDM status, a similar pattern of results was observed in normoglycaemic and GDM cases (Table 3). Of note, among the GDM cases, differences in the magnitude (beta coefficient) of the glycaemic-birthweight associations between the lowest/middle inositol tertiles and the highest inositol tertile were more marked than those seen among the normoglycaemic cases.

In the subset of infants with MRI data, each mmol/L rise in FPG was associated with increasing sSAT (β = 10.5 ml, 95% CI: 6.7–14.3), dSAT (β = 2.8 ml, 95% CI: 1.6–4.0), IAT (β = 3.9 ml, 95% CI: 2.4–5.4) and TAAT (β = 17.3 ml, 95% CI: 11.9–22.6) volumes. For each of these adipose tissue compartments, there were similar effect sizes (β) in both the lowest and middle inositol tertiles without (Fig. 1b illustrating TAAT) and with covariate adjustments (Table 4). Similar to birthweight and birthweight z-score, no association was observed within the highest inositol tertile. Among normoglycaemic cases, the positive association between FPG and AAT compartment volumes was only observed within the lowest inositol tertile but not in the middle and highest tertiles (Table 4). Analysis was not conducted for GDM cases due to inadequate sample size.

Sensitivity analyses (with exclusion of possible pre-existing diabetes cases [FPG ≥ 7.0 mmol/L or 2hPG ≥ 11.1 mmol/L], mothers who received insulin treatment for GDM, hypertensive disorders, and small-for-gestational-age infants) yielded similar results for birthweight, birthweight z-score and AAT (Electronic Supplementary Material [ESM] Tables 1–8). Of note, a clearer gradation of effect could now be observed for the association between FPG and AAT volumes across the placental inositol tertiles after removing possible pre-existing diabetes cases (ESM Table 2). For TAAT, the greatest effect size was observed among cases in the lowest inositol tertile (β = 22.9 ml; 95% CI: 13.4–32.5), a less marked and no longer significant effect in the middle tertile (β = 8.2 ml; 95% CI: −5.5–21.8), and no effect in the highest tertile (β = 0.8 ml; 95% CI: −8.4–10.0). Further, with technical adjustment of inositol values for mode of delivery and timing of placental collection where data was available (*n* = 684), results remained similar (ESM Tables 9 and 10).

No significant interactions were noted between inositol and ethnicity or neonatal sex in relation to birthweight and adiposity outcomes.

## Discussion

This large novel study of human placental inositol quantification demonstrated that a lower placental inositol content was associated with higher maternal 2hPG, no exposure to tobacco smoke antenatally, with vaginal delivery and shorter gestation. A positive association between maternal glycaemia and birthweight or offspring abdominal adiposity was only observed among cases of low, but not high, placental inositol levels. This is consistent with our hypothesis that a high placental inositol content could attenuate the pro-adipogenic effects of increasing maternal glycaemia in the offspring. This suggests a potentially innovative application for inositol supplementation in pregnancy to mitigate excessive fetal adiposity risk, and the subsequent effects on long-term metabolic health. It is important to note that none of the women in this cohort reported taking antenatal supplements containing inositol.

The observed decrease in placental inositol content with increasing levels of antenatal 2hPG may result from a combination of reduced local placental cellular uptake of inositol (due to competitive inhibition of membrane transporters by glucose), enhanced inositol efflux (secondary to sorbitol accumulation), altered inositol synthesis and increased catabolism, just as for other tissues in non-pregnant type 1 and 2 diabetes cases [14].

It is unclear how tobacco smoke exposure could be associated with increased placental inositol. In a metabolomics study of non-pregnant individuals, circulating levels of lipid-conjugated scyllo-inositol, one of the less abundant inositol isomers, was negatively associated with smoking [25]. It may suggest that tobacco smoke exposure alters the balance of lipid-conjugated and water-soluble inositol, such that water-soluble inositol in the placenta is increased with tobacco smoke exposure. We observed that placental inositol was positively associated with gestational age, although the cause-effect direction of gestational age and placental inositol remains unclear. Nonetheless, our result is consistent with an RCT showing that women receiving inositol supplementation had a longer gestation [13]. Vaginal delivery was significantly associated with reduced placental inositol. It is possible that undergoing the labour process *per se* reduced placental inositol, since a milder but insignificant reduction in placental inositol was seen in those who did not complete the labour process (but had an intrapartum caesarean section). We cannot, however, exclude the possibility that placental inositol may be involved in modulating the onset and progress of labour. Nevertheless, the analyses of only cases of spontaneous onset of labour showed no significant associations between placental inositol at delivery and gestational age or mode of delivery (data not shown).

Previous publications have shown that maternal FPG is more strongly associated with neonatal adiposity than post-load glycaemia [6, 26]. Our results highlight the potential for high placental inositol to suppress the effects of maternal FPG in the promotion of neonatal birthweight and adiposity across all AAT compartments. This could be clinically relevant since central adiposity, especially dSAT, is particularly linked with metabolic adversity of insulin resistance and pro-inflammation and increased cardiovascular risks [27].

Emerging evidence suggests that inositol supplementation could be useful in preventing GDM. Given that such an approach would lead to a widespread introduction of inositol supplementation that would expose many pregnancies to additional exogenous inositol (including those that would not ultimately develop GDM), it is critical that we improve our understanding of inositol’s impact on fetoplacental physiology. Some trials documented lower birthweight with inositol supplementation in metabolically at risk women [7, 13], but our results from this predominantly metabolically healthy cohort showed no association between placental inositol and birthweight.

Our results suggest that inositols may have effects on neonatal adiposity through mechanisms that are distinct from those directly related to maternal glycaemic regulation. Assuming a high placental inositol level assessed at delivery is reflective of the systemic inositol levels present antenatally, this did not necessarily avert the occurrence of maternal hyperglycaemia in our cohort as would be expected from inositol promotion of insulin sensitivity in maternal tissues [15]. Even at a high level of placental inositol, we still observed maternal FPG readings of up to 6.5 mmol/L. However, a high placental inositol level was associated with the abolition of the glycaemia-associated increase in birthweight and abdominal adiposity. We speculate that this effect could arise through the inositol modulation of placental functions including lipid flux and metabolism [28], and many second messenger signalling pathways [29] with consequences on fetal adiposity. Indeed, inositol treatment in vitro has been shown to affect these processes in human placenta [28] and in several non-placental tissues [14, 29].

The strengths of this study include a large sample size of placentae having inositol levels reliably measured by LC-MS, robust measurements of neonatal abdominal adiposity using MRI, the prospective clinical data collection, and the series of sensitivity analyses ascertaining the robustness of results. The study has several limitations. As placental inositol content could only be assessed at the point of delivery, with the lack of an inositol measure during pregnancy, this hindered the causal inferences that can be drawn about inositol’s modulatory role during fetal development. Such a limitation is true of placental studies in general and it is acceptable in this field to assume that levels measured at delivery are reflective of those in utero during fetal development. It also cannot be determined if the variation in placenta inositol content was reflective of the differences in systemic levels of inositol which could potentially be addressed through supplementation, or a result of altered local placental uptake and metabolic processes. Other limitations include the lack of resolution of the full range of nine inositol isomers, and that only the water-soluble inositols, not the lipid-conjugated components, were quantified. We cannot exclude residual confounding by other factors, including other lifestyle and genetic variations, that may be associated with differences in placental inositol content which could concomitantly affect the relation between maternal glycaemia and fetal adiposity. Lastly, our findings may not necessarily be generalised beyond Asian Singaporeans.

Our results support the hypothesis that a high placental inositol content may mitigate the pro-adipogenic effects of maternal glycaemia, resulting in lower birthweight and adiposity in the offspring; this being independent of the role of inositol in maternal tissues to improve glucose metabolism. Further research is needed to test if inositol supplementation in pregnancy can increase placental inositol content [30] and reduce fetal adiposity associated with increasing maternal glycaemia. This would represent a novel application for inositol supplementation.

## Supplementary information

Supplementary information

## Data Availability

Data is available upon request from the GUSTO team.

## References

[1] Farrar D, Simmonds M, Bryant M, Sheldon TA, Tuffnell D, Golder S (2016). Hyperglycaemia and risk of adverse perinatal outcomes: systematic review and meta-analysis. BMJ.

[2] Kerényi Z, Tamás G, Kivimäki M, Péterfalvi A, Madarász E, Bosnyák Z (2009). Maternal glycemia and risk of large-for-gestational-age babies in a population-based screening. Diabetes Care.

[3] Metzger BE, Contreras M, Sacks DA, Watson W, Dooley SL, Foderaro M (2008). Hyperglycemia and adverse pregnancy outcomes. New Engl J Med.

[4] Lowe WL, Lowe LP, Kuang A, Catalano PM, Nodzenski M, Talbot O (2019). Maternal glucose levels during pregnancy and childhood adiposity in the Hyperglycemia and Adverse Pregnancy Outcome Follow-up Study. Diabetologia.

[5] Pedersen J (1952). Diabetes and pregnancy; blood sugar of newborn infants during fasting and glucose administration. Nordisk Med.

[6] Catalano PM, Thomas A, Huston-Presley L, Amini SB (2003). Increased fetal adiposity: a very sensitive marker of abnormal in utero development. Am J Obstet Gynecol.

[7] D’Anna R, Scilipoti A, Giordano D, Caruso C, Cannata ML, Interdonato ML (2013). myo-Inositol supplementation and onset of gestational diabetes mellitus in pregnant women with a family history of type 2 diabetes: a prospective, randomized, placebo-controlled study. Diabetes Care.

[8] D’anna R, Di Benedetto V, Rizzo P, Raffone E, Interdonato ML, Corrado F (2012). Myo-inositol may prevent gestational diabetes in PCOS women. Gynecol Endocrinol.

[9] Santamaria A, Di Benedetto A, Petrella E, Pintaudi B, Corrado F, D’Anna R (2016). Myo-inositol may prevent gestational diabetes onset in overweight women: a randomized, controlled trial. J Matern Fetal Neonatal Med.

[10] D’Anna R, Di Benedetto A, Scilipoti A, Santamaria A, Interdonato ML, Petrella E (2015). Myo-inositol supplementation for prevention of gestational diabetes in obese pregnant women: a randomized controlled trial. Obstet Gynecol.

[11] Farren M, Daly N, McKeating A, Kinsley B, Turner MJ, Daly S (2017). The prevention of gestational diabetes mellitus with antenatal oral inositol supplementation: a randomized controlled trial. Diabetes Care.

[12] Pintaudi B, Di Vieste G (2017). Comment on Farren et al. The prevention of gestational diabetes mellitus with antenatal oral inositol supplementation: a randomized controlled trial. Diabetes Care.

[13] Matarrelli B, Vitacolonna E, D’angelo M, Pavone G, Mattei PA, Liberati M (2013). Effect of dietary myo-inositol supplementation in pregnancy on the incidence of maternal gestational diabetes mellitus and fetal outcomes: a randomized controlled trial. J Matern Fetal Neonatal Med.

[14] Holub BJ (1986). Metabolism and function of myo-inositol and inositol phospholipids. Annu Rev Nutr.

[15] Coustan DR (2013). Can a dietary supplement prevent gestational diabetes mellitus?. Diabetes Care.

[16] Staat BC, Galan HL, Harwood JE, Lee G, Marconi AM, Paolini CL (2012). Transplacental supply of mannose and inositol in uncomplicated pregnancies using stable isotopes. J Clin Endocrinol Metab.

[17] Toh N, Inoue T, Tanaka H, Kimoto E (1987). Polyol accumulation in human placenta and umbilical cord. Biol Res Pregnancy Perinatol.

[18] Soh SE, Tint MT, Gluckman PD, Godfrey KM, Rifkin-Graboi A, Chan YH (2014). Cohort profile: Growing Up in Singapore Towards healthy Outcomes (GUSTO) birth cohort study. Int J Epidemiol.

[19] WHO Expert Consultation. (2004). Appropriate body-mass index for Asian populations and its implications for policy and intervention strategies. Lancet.

[20] Ng S, Aris IM, Tint MT, Gluckman PD, Godfrey KM, Shek LP (2019). High maternal circulating cotinine during pregnancy is associated with persistently shorter stature from birth to five years in an Asian cohort. Nicotine Tob Res.

[21] Hadlock FP, Shah YP, Kanon DJ, Lindsey JV (1992). Fetal crown-rump length: reevaluation of relation to menstrual age (5-18 weeks) with high-resolution real-time US. Radiology.

[22] Mikolajczyk RT, Zhang J, Betran AP, Souza JP, Mori R, Gülmezoglu AM (2011). A global reference for fetal-weight and birthweight percentiles. Lancet.

[23] Tint MT, Fortier MV, Godfrey KM, Shuter B, Kapur J, Rajadurai VS (2016). Abdominal adipose tissue compartments vary with ethnicity in Asian neonates: Growing Up in Singapore Toward Healthy Outcomes birth cohort study. Am J Clin Nutr.

[24] Stewart RJ, Whitehead L, Nijagal B, Sleebs BE, Lessene G, McConville MJ (2017). Analysis of Ca(2)(+) mediated signaling regulating Toxoplasma infectivity reveals complex relationships between key molecules. Cell Microbiol.

[25] Cross AJ, Boca S, Freedman ND, Caporaso NE, Huang WY, Sinha R (2014). Metabolites of tobacco smoking and colorectal cancer risk. Carcinogenesis.

[26] Aris IM, Soh SE, Tint MT, Liang S, Chinnadurai A, Saw SM (2014). Effect of maternal glycemia on neonatal adiposity in a multiethnic Asian birth cohort. J Clin Endocrinol Metab.

[27] Pi-Sunyer FX (2004). The epidemiology of central fat distribution in relation to disease. Nutr Rev.

[28] Watkins OC, Islam MO, Selvam P, Pillai RA, Cazenave-Gassiot A (2019). Myo-inositol alters 13C-labeled fatty acid metabolism in human placental explants. J Endocrinol.

[29] Croze ML, Soulage CO (2013). Potential role and therapeutic interests of myo-inositol in metabolic diseases. Biochimie.

[30] Godfrey KM, Cutfield W, Chan SY, Baker PN, Chong YS, NiPPeR Study Group. (2017). Nutritional Intervention Preconception and During Pregnancy to Maintain Healthy Glucose Metabolism and Offspring Health (“NiPPeR”): study protocol for a randomised controlled trial. Trials.

