# Supplementary material for: High placental inositol content associated with suppressed pro-adipogenic effects of maternal glycaemia in offspring: the GUSTO cohort
Source: Int J Obes (Lond). 2020 May 20;45(1):247–57. doi: 10.1038/s41366-020-0596-5 (PMC7752752; doi:10.1038/s41366-020-0596-5)
Supplement: Supplementary file 1 — Supplementary information [file 41366_2020_596_MOESM1_ESM.docx]

**Electronic Supplementary Material**

**ESM Table 1.** Adjusted associations between maternal mid-gestation FPG and birthweight stratified by placental inositol tertiles after exclusion of possible pre-existing diabetes cases (FPG ≥7.0 mmol/L or 2hPG ≥11.1 mmol/L; n=6).

| **FPG (mmol/L)** | **Birthweight (g)^†^** |  | **Birthweight z-scores (SDs)^‡^** |  |
| --- | --- | --- | --- | --- |
| **Tertiles of inositol** | **β**^§^ **(95% CI)** | ***P* value** | **β**^§^ **(95% CI)** | ***P* value** |
| **Lowest (n=241^‖^)** | 182.4 (74.5, 290.2) | 0.001 | 0.6 (0.3, 1) | 0.001 |
| **Middle (n=238^‖^)** | 181.5 (69.3, 293.7) | 0.002 | 0.6 (0.2, 0.9) | 0.002 |
| **Highest (n=239^‖^)** | 81.0 (-21.2, 183.2) | 0.120 | 0.2 (-0.1, 0.5) | 0.242 |

**^†^**Adjusted for maternal age, ethnicity, parity, pre-pregnancy BMI, tobacco smoke exposure, gestational age, and neonatal sex.

**^‡^**Adjusted for maternal age, ethnicity, parity, pre-pregnancy BMI, and tobacco smoke exposure.
^§^Beta (β) represents the change in g or SDs per mmol/L increase in FPG.
**^‖^**Only cases with full data sets available are presented.
CI, confidence interval; FPG, fasting plasma glucose.

**ESM Table 2.** Adjusted associations between maternal mid-gestation FPG and neonatal abdominal adiposity stratified by placental inositol tertiles after exclusion of possible pre-existing diabetes cases (FPG ≥7.0 mmol/L or 2hPG ≥11.1 mmol/L; n=4).

| **FPG (mmol/L)** | **sSAT (ml)** |  | **dSAT (ml)** |  | **IAT (ml)** |  | **TAAT (ml)** |  |
| --- | --- | --- | --- | --- | --- | --- | --- | --- |
| **Tertiles of inositol** | **β**^§^ **(95% CI)** | ***P* value** | **β**^§^ **(95% CI)** | ***P* value** | **β**^§^ **(95% CI)** | ***P* value** | **β**^§^ **(95% CI)** | ***P* value** |
| **Lowest (n=78^‖^)** | 13.4  (6.6, 20.2) | <0.001 | 4.0  (1.7, 6.2) | 0.001 | 5.6  (3.0, 8.1) | <0.001 | 22.9  (13.4, 32.5) | <0.001 |
| **Middle (n=63^‖^)** | 5.3  (-3.6, 14.2) | 0.236 | 2.1  (-1.0, 5.2) | 0.184 | 0.8  (-3.5, 5.1) | 0.715 | 8.2  (-5.5, 21.8) | 0.234 |
| **Highest (n=74^‖^)** | 1.6  (-4.9, 8.0) | 0.625 | -0.3  (-2.6, 2.0) | 0.805 | -0.5  (-3.9, 2.9) | 0.865 | 0.8  (-8.4, 10.0) | 0.862 |

Adjusted for maternal age, ethnicity, parity, pre-pregnancy BMI, tobacco smoke exposure, gestational age, neonatal sex, birthweight, and age on MRI day.

^§^Beta (β) represents the change in ml per mmol/L increase in FPG.
**^‖^**Only cases with full data sets available are presented.

CI, confidence interval; dSAT, deep subcutaneous adipose tissue; FPG, fasting plasma glucose; IAT, internal adipose tissue; sSAT, superficial subcutaneous adipose tissue; TAAT, total abdominal adipose tissue.

**ESM Table 3.** Adjusted associations between maternal mid-gestation FPG and birthweight stratified by placental inositol tertiles excluding mothers on insulin treatment for GDM (encompassing those with possible pre-existing diabetes; n=10).

| **FPG (mmol/L)** | **Birthweight (g)^†^** |  | **Birthweight z-scores (SDs)^‡^** |  |
| --- | --- | --- | --- | --- |
| **Tertiles of inositol** | **β**^§^ **(95% CI)** | ***P* value** | **β**^§^ **(95% CI)** | ***P* value** |
| **Lowest (n=241^‖^)** | 181 (51.3, 310.7) | <0.001 | 0.6 (0.3, 1) | 0.001 |
| **Middle (n=238^‖^)** | 152.4 (46.4, 258.4) | <0.001 | 0.6 (0.3, 1) | <0.001 |
| **Highest (n=235^‖^)** | 73.4 (-43.7, 190.6) | 0.200 | 0.2 (-0.2, 0.6) | 0.264 |

**^†^**Adjusted for maternal age, ethnicity, parity, pre-pregnancy BMI, tobacco smoke exposure, gestational age, and neonatal sex.

**^‡^**Adjusted for maternal age, ethnicity, parity, pre-pregnancy BMI, and tobacco smoke exposure.
^§^Beta (β) represents the change in g or SDs per mmol/L increase in FPG.
**^‖^**Only cases with full data sets available are presented.
CI, confidence interval; FPG, fasting plasma glucose.

**ESM Table 4.** Adjusted associations between maternal mid-gestation FPG and neonatal abdominal adiposity stratified by placental inositol tertiles excluding mothers on insulin treatment for GDM (encompassing those with possible pre-existing diabetes; n=5).

| **FPG (mmol/L)** | **sSAT (ml)** |  | **dSAT (ml)** |  | **IAT (ml)** |  | **TAAT (ml)** |  |
| --- | --- | --- | --- | --- | --- | --- | --- | --- |
| **Tertiles of inositol** | **β**^§^ **(95% CI)** | ***P* value** | **β**^§^ **(95% CI)** | ***P* value** | **β**^§^ **(95% CI)** | ***P* value** | **β**^§^ **(95% CI)** | ***P* value** |
| **Lowest (n=78^‖^)** | 12.7  (5.1, 20.3) | 0.001 | 3.6  (1.3, 5.9) | 0.003 | 6.1  (3.5, 8.6) | <0.001 | 22.4  (12.1, 32.7) | <0.001 |
| **Middle (n=64^‖^)** | 12.0  (5.5, 18.5) | 0.001 | 3.2  (1, 5.4) | 0.005 | 4.0  (1, 7) | 0.01 | 19.2  (9.2, 29.1) | <0.001 |
| **Highest (n=72^‖^)** | -1.2  (-9.1, 6.7) | 0.758 | -1  (-3.9, 1.9) | 0.492 | -2.0  (-6.2, 2.2) | 0.337 | -4.2  (-15.4, 6.9) | 0.453 |

Adjusted for maternal age, ethnicity, parity, pre-pregnancy BMI, tobacco smoke exposure, gestational age, neonatal sex, birthweight, and age on MRI day.

^§^Beta (β) represents the change in ml per mmol/L increase in FPG.
**^‖^**Only cases with full data sets available are presented.

CI, confidence interval; dSAT, deep subcutaneous adipose tissue; FPG, fasting plasma glucose; IAT, internal adipose tissue; sSAT, superficial subcutaneous adipose tissue; TAAT, total abdominal adipose tissue.

**ESM Table 5.** Adjusted associations between maternal mid-gestation FPG and birthweight stratified by placental inositol tertiles after removal of cases with hypertensive disorders of pregnancy and chronic hypertension (n=44).

| **FPG (mmol/L)** | **Birthweight (g)^†^** |  | **Birthweight z-scores (SDs)^‡^** |  |
| --- | --- | --- | --- | --- |
| **Tertiles of inositol** | **β**^§^ **(95% CI)** | ***P* value** | **β**^§^ **(95% CI)** | ***P* value** |
| **Lowest (n=232^‖^)** | 152.1 (47.6, 256.7) | 0.005 | 0.5 (0.2, 0.8) | 0.004 |
| **Middle (n=226^‖^)** | 205.0 (107.8, 302.3) | <0.001 | 0.7 (0.3, 1) | <0.001 |
| **Highest (n=222^‖^)** | 85.4 (-32.1, 202.9) | 0.153 | 0.2 (-0.2, 0.6) | 0.314 |

**^†^**Adjusted for maternal age, ethnicity, parity, pre-pregnancy BMI, tobacco smoke exposure, gestational age, and neonatal sex.

**^‡^**Adjusted for maternal age, ethnicity, parity, pre-pregnancy BMI, and tobacco smoke exposure.
^§^Beta (β) represents the change in g or SDs per mmol/L increase in FPG.
**^‖^**Only cases with full data sets available are presented.
CI, confidence interval; FPG, fasting plasma glucose.

**ESM Table 6.** Adjusted associations between maternal mid-gestation FPG and neonatal abdominal adiposity stratified by placental inositol tertiles after removal of cases with hypertensive disorders of pregnancy and chronic hypertension (n=14).

| **FPG (mmol/L)** | **sSAT (ml)** |  | **dSAT (ml)** |  | **IAT (ml)** |  | **TAAT (ml)** |  |
| --- | --- | --- | --- | --- | --- | --- | --- | --- |
| **Tertiles of inositol** | **β**^§^ **(95% CI)** | ***P* value** | **β**^§^ **(95% CI)** | ***P* value** | **β**^§^ **(95% CI)** | ***P* value** | **β**^§^ **(95% CI)** | ***P* value** |
| **Lowest (n=66^‖^)** | 9.7  (1.8, 17.6) | 0.017 | 3.2  (0.8, 5.5) | 0.009 | 5.8  (3.0, 8.6) | <0.001 | 18.7  (8.0, 29.3) | 0.001 |
| **Middle (n=59^‖^)** | 11.9  (5.2, 18.7) | 0.001 | 3.2  (0.9, 5.5) | 0.008 | 4.4  (1.0, 7.8) | 0.011 | 19.5  (9.0, 30.1) | <0.001 |
| **Highest (n=67^‖^)** | -0.7  (-8.9, 7.5) | 0.864 | -0.8  (-3.8, 2.2) | 0.602 | -1.9  (-6.4, 2.5) | 0.386 | -3.4  (-15.1, 8.3) | 0.564 |

Adjusted for maternal age, ethnicity, parity, pre-pregnancy BMI, tobacco smoke exposure, gestational age, neonatal sex, birthweight, and age on MRI day.

^§^Beta (β) represents the change in ml per mmol/L increase in FPG.
**^‖^**Only cases with full data sets available are presented.

CI, confidence interval; dSAT, deep subcutaneous adipose tissue; FPG, fasting plasma glucose; IAT, internal adipose tissue; sSAT, superficial subcutaneous adipose tissue; TAAT, total abdominal adipose tissue.

**ESM Table 7.** Adjusted associations between maternal mid-gestation FPG and birthweight stratified by placental inositol tertiles after removal of small-for-gestational-age infants (<10^th^ centile; n=73).

| **FPG (mmol/L)** | **Birthweight (g)^†^** |  | **Birthweight z-scores (SDs)^‡^** |  |
| --- | --- | --- | --- | --- |
| **Tertiles of inositol** | **β**^§^ **(95% CI)** | ***P* value** | **β**^§^ **(95% CI)** | ***P* value** |
| **Lowest (n=216^‖^)** | 152.1 (70.7, 233.4) | <0.001 | 0.5 (0.3, 0.8) | <0.001 |
| **Middle (n=214^‖^)** | 166.9 (74.4, 259.4) | <0.001 | 0.5 (0.2, 0.8) | 0.001 |
| **Highest (n=221^‖^)** | 67.7 (-22.9, 158.4) | 0.142 | 0.2 (-0.1, 0.4) | 0.307 |

**^†^**Adjusted for maternal age, ethnicity, parity, pre-pregnancy BMI, tobacco smoke exposure, gestational age, and neonatal sex.

**^‡^**Adjusted for maternal age, ethnicity, parity, pre-pregnancy BMI, and tobacco smoke exposure.
^§^Beta (β) represents the change in g or SDs per mmol/L increase in FPG.
**^‖^**Only cases with full data sets available are presented.
CI, confidence interval; FPG, fasting plasma glucose.

**ESM Table 8.** Adjusted associations between maternal mid-gestation FPG and neonatal abdominal adiposity stratified by placental inositol tertiles after removal of small-for-gestational-age infants (<10^th^ centile; n=27).

| **FPG (mmol/L)** | **sSAT (ml)** |  | **dSAT (ml)** |  | **IAT (ml)** |  | **TAAT (ml)** |  |
| --- | --- | --- | --- | --- | --- | --- | --- | --- |
| **Tertiles of inositol** | **β**^§^ **(95% CI)** | ***P* value** | **β**^§^ **(95% CI)** | ***P* value** | **β**^§^ **(95% CI)** | ***P* value** | **β**^§^ **(95% CI)** | ***P* value** |
| **Lowest (n=66^‖^)** | 11.9  (5.3, 18.5) | 0.001 | 3.5  (1.5, 5.5) | 0.001 | 5.1  (2.9, 7.3) | <0.001 | 20.5  (11.7, 29.2) | <0.001 |
| **Middle (n=59^‖^)** | 13.0  (6.4, 19.5) | <0.001 | 3.8  (1.4, 6.2) | 0.002 | 5.3  (2.1, 8.4) | 0.002 | 22.0  (11.9, 32.2) | <0.001 |
| **Highest (n=67^‖^)** | 1.3  (-5.5, 8.2) | 0.700 | -2.4  (-2.6, 2.2) | 0.841 | -0.1  (-3.5, 3.2) | 0.934 | 0.9  (-8.7, 10.6) | 0.844 |

Adjusted for maternal age, ethnicity, parity, pre-pregnancy BMI, tobacco smoke exposure, gestational age, neonatal sex, birthweight, and age on MRI day.

^§^Beta (β) represents the change in ml per mmol/L increase in FPG.
**^‖^**Only cases with full data sets available are presented.

CI, confidence interval; dSAT, deep subcutaneous adipose tissue; FPG, fasting plasma glucose; IAT, internal adipose tissue; sSAT, superficial subcutaneous adipose tissue; TAAT, total abdominal adipose tissue.

**ESM Table 9.** Associations between maternal mid-gestation FPG and birthweight stratified by placental inositol tertiles – with both confounder adjustments and technical adjustments of placental inositol values for the peri-partum factor of mode of delivery and pre-analytical factor of timing of placental collection.

| **FPG (mmol/L)** | **Birthweight (g)^†^** |  | **Birthweight z-scores (SDs)^‡^** |  |
| --- | --- | --- | --- | --- |
| **Tertiles of inositol** | **β**^§^ **(95% CI)** | ***P* value** | **β**^§^ **(95% CI)** | ***P* value** |
| **Lowest (n=231^‖^)** | 168.4 (72.4, 264.5) | 0.001 | 0.6 (0.3, 0.9) | <0.001 |
| **Middle (n=226^‖^)** | 211.9 (115.7, 308.1) | <0.001 | 0.7 (0.4, 1.0) | <0.001 |
| **Highest (n=227^‖^)** | 104.9 (-12.2, 221.9) | 0.079 | 0.3 (-0.1, 0.6) | 0.169 |

**^†^**Adjusted for maternal age, ethnicity, parity, pre-pregnancy BMI, tobacco smoke exposure, gestational age, neonatal sex, mode of delivery and timing of placental collection.

**^‡^**Adjusted for maternal age, ethnicity, parity, pre-pregnancy BMI, and tobacco smoke exposure.
^§^Beta (β) represents the change in g or SDs per mmol/L increase in FPG.
**^‖^**Only cases with full data sets available are presented.
CI, confidence interval; FPG, fasting plasma glucose.

**ESM Table 10.** Associations between maternal mid-gestation FPG and neonatal abdominal adiposity stratified by placental inositol tertiles – with both confounder adjustments and technical adjustments of placental inositol values for the peri-partum factor of mode of delivery and pre-analytical factor of timing of placental collection.

| **FPG (mmol/L)** | **sSAT (ml)** |  | **dSAT (ml)** |  | **IAT (ml)** |  | **TAAT (ml)** |  |
| --- | --- | --- | --- | --- | --- | --- | --- | --- |
| **Tertiles of inositol** | **β**^§^ **(95% CI)** | ***P* value** | **β**^§^ **(95% CI)** | ***P* value** | **β**^§^ **(95% CI)** | ***P* value** | **β**^§^ **(95% CI)** | ***P* value** |
| **Lowest (n=75^‖^)** | 13.0  (7.0, 18.9) | <0.001 | 3.6  (1.9, 5.4) | <0.001 | 5.0  (3.1, 7.0) | <0.001 | 21.6  (13.7, 29.6) | <0.001 |
| **Middle (n=59^‖^)** | 12.6  (5.6, 19.7) | 0.001 | 2.6  (0.2, 4.9) | 0.034 | 4.3  (1, 7.7) | 0.013 | 19.5  (8.6, 30.4) | 0.001 |
| **Highest (n=69^‖^)** | 0.1  (-7.2, 7.4) | 0.976 | -0.6  (-3.4, 2.3) | 0.698 | -2.8  (-7.1, 1.5) | 0.200 | -3.2  (-14.3, 7.9) | 0.564 |

Adjusted for maternal age, ethnicity, parity, pre-pregnancy BMI, tobacco smoke exposure, gestational age, neonatal sex, birthweight, age on MRI day, mode of delivery and timing of placental collection
^§^Beta (β) represents the change in ml per mmol/L increase in FPG.
**^‖^**Only cases with full data sets available are presented.
CI, confidence interval; dSAT, deep subcutaneous adipose tissue; FPG, fasting plasma glucose; IAT, internal adipose tissue; sSAT, superficial subcutaneous adipose tissue; TAAT, total abdominal adipose tissue.
